# Annexins and cardiovascular diseases: Beyond membrane trafficking and repair

**DOI:** 10.3389/fcell.2022.1000760

**Published:** 2022-10-14

**Authors:** Nerea Méndez-Barbero, Irene San Sebastian-Jaraba, Rafael Blázquez-Serra, Jose L. Martín-Ventura, Luis M. Blanco-Colio

**Affiliations:** ^1^ Laboratory of Vascular Pathology, IIS-Fundación Jiménez Díaz, Madrid, Spain; ^2^ CIBERCV, Madrid, Spain; ^3^ Autonoma University of Madrid, Madrid, Spain

**Keywords:** annexin, cardiovascular diseases, ischemic event, atherosclerosis, inflammation

## Abstract

Cardiovascular diseases (CVD) remain the leading cause of mortality worldwide. The main cause underlying CVD is associated with the pathological remodeling of the vascular wall, involving several cell types, including endothelial cells, vascular smooth muscle cells, and leukocytes. Vascular remodeling is often related with the development of atherosclerotic plaques leading to narrowing of the arteries and reduced blood flow. Atherosclerosis is known to be triggered by high blood cholesterol levels, which in the presence of a dysfunctional endothelium, results in the retention of lipoproteins in the artery wall, leading to an immune-inflammatory response. Continued hypercholesterolemia and inflammation aggravate the progression of atherosclerotic plaque over time, which is often complicated by thrombus development, leading to the possibility of CV events such as myocardial infarction or stroke. Annexins are a family of proteins with high structural homology that bind phospholipids in a calcium-dependent manner. These proteins are involved in several biological functions, from cell structural organization to growth regulation and vesicle trafficking. *In vitro* gain- or loss-of-function experiments have demonstrated the implication of annexins with a wide variety of cellular processes independent of calcium signaling such as immune-inflammatory response, cell proliferation, migration, differentiation, apoptosis, and membrane repair. In the last years, the use of mice deficient for different annexins has provided insight into additional functions of these proteins *in vivo*, and their involvement in different pathologies. This review will focus in the role of annexins in CVD, highlighting the mechanisms involved and the potential therapeutic effects of these proteins.

## Introduction

Cardiovascular diseases (CVD) are the leading cause of mortality in developed countries. Although the death rate due to cardiovascular causes has decreased considerably in the last two decades, 17.3 million people die annually from CVD and it is estimated that this will increase to 23.6 million people by 2030 (WHO CVD Risk Chart Working Group, 2019). The term CVD is used to describe different pathologies affecting the heart and circulatory system, including heart failure, coronary artery disease, stroke, hypertension and atherosclerosis, among others. In particular, atherosclerosis, considered the major precursor of CVD, refers to the development of atheromatous plaques in the inner lining of medium and large arteries. Resting endothelium is, generally, resistant to leukocyte binding. In an atherogenic environment, different stimuli such as hyperlipidemia, hypertension or diabetes induces the expression of adhesion molecules by endothelial cells that allow anchoring of white blood cells to the intimal surface. The expression of different chemokines directs the migration of leukocytes into the arterial wall. Within the arterial wall, mononuclear phagocytes engulf lipids and differentiate into lipid-loaded foam cells, a hallmark of atherosclerosis. Recent linage-tracking experiments have also revealed a smooth muscle origin of many foam cells in atherosclerotic plaques of mice (Bennett et al., 2016). In addition, a proinflammatory subset of monocytes differentiate to lesional macrophages (Tacke et al., 2007) and T lymphocytes interact with innate immune cells cooperating to increase proinflammatory cytokines expression that amplify and perpetuate the inflammatory response. The concerted action of pro-inflammatory signals hampers renewal of the structural elements that support the stability of plaques.

The intimal layer also contains resident smooth muscle cells (SMCs) that will proliferate and, together with the SMCs that have migrated from the media, produce extracellular matrix proteins such as interstitial collagen and elastin, which promote the formation of the fibrous cap that covers the plaque. This layer normally overlaps foam cells, which may die and release lipids that accumulate extracellularly. Ineffective removal of dead cells, a process known as efferocytosis, can result in the accumulation of cellular debris and extracellular lipids forming the necrotic core (Tabas, 2010). Thinning of the fibrous cap arises from a decrease in collagen synthesis and increased degradation by overexpression of collagenases by inflammatory cells (Libby, 2013).

Atherosclerotic plaque generally causes clinical manifestations derived from the stenosis it produces in the vessel lumen, limiting flow and resulting in ischemic tissue, or by causing thrombi that can interrupt blood flow locally. The rupture of the fibrous cap exposes the procoagulant material in the plaque core to blood clotting proteins, which triggers thrombosis. Thrombus formation in the artery will lead to myocardial infarction, stroke, unstable angina or sudden death (Hansson et al., 2015).

### Annexins A, a family of proteins implicated in membrane trafficking and repair

**FIGURE 1:**
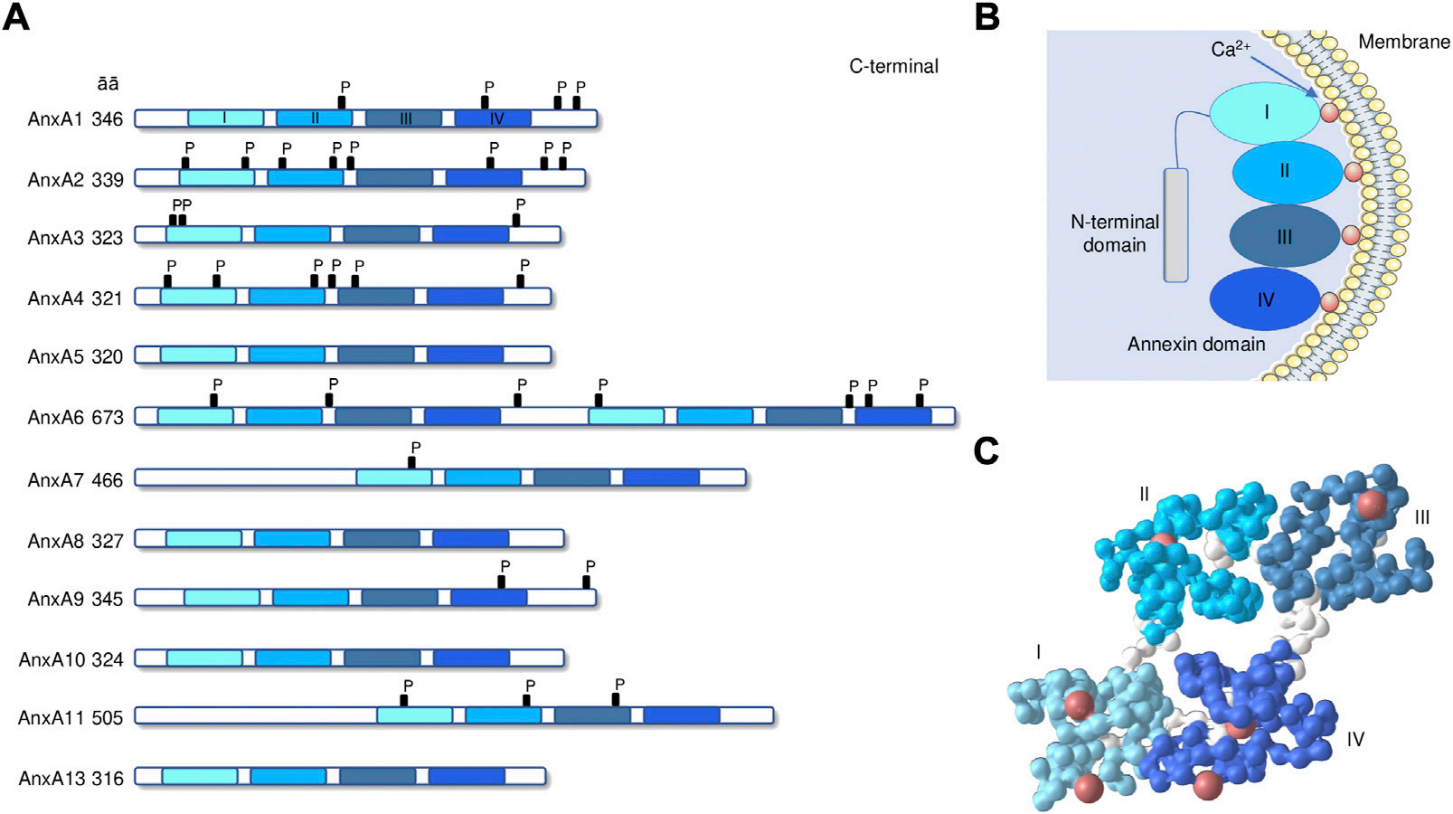
Annexin structure. **(A)** The domain structures of annexins AnxA1 to A13 are illustrated. C-terminal core with annexin repeats I–IV in light to dark blue and length (in amino acids) are indicated. P, known phosphorylation sites. **(B)** Schematic drawing of annexin peripherally attached to a membrane surface through bound Ca2+ ions. **(C)** 3D-structure of human AnxA1 (PDB: 1AIN [Weng et al., 1993]). Colors identify the 4 repeated domains (spacer regions in white), with coordinated calcium ions (red). 3D-structures are visualized with the iCn3D software vs. 3.15.1 (https://www.ncbi.nlm.nih.gov/Structure/icn3d/icn3d.html).

Annexins constitute a multigene family of Ca^2+^-dependent phospholipid-binding proteins with members of the family being expressed in animals, plants, fungi, and protists (Gerke and Moss, 2002). This family can be divided into five classes (A-E) based on their biological origins. In vertebrates, the class A is composed by 12 members referred to as AnxA1-AnxA13 (AnxA12 is currently unassigned) (Gerke and Moss, 2002). Annexins have two structural domains: a conserved C-terminal domain with 4 repeats of 70–80 amino acids (8 in the case of AnxA6) (Figure 1A) and a variable N-terminal domain. Each C-terminal domain repeats of annexins includes a Ca^2+^-binding motif, allowing the annexins to rapidly translocate to the plasma membrane or intracellular membranes by binding to negatively charged phospholipids (Gerke et al., 2005; Grewal and Enrich, 2009) (Figure 1B). Their functions are dependent on their dynamic and reversible membrane binding behavior. The N-terminal domain is highly variable in sequence and length and confers diverse functions to the different members of the family (Rescher and Gerke, 2004). Different *in vitro* studies with purified annexins have analyzed their biochemical properties, their folding and 3D structure (Figure 1C), as well as their membrane binding characteristics and their affinities for different membrane phospholipids and with other proteins, particularly the S100 family of proteins (Weisz and Uversky, 2020). Annexins are found at multiple locations inside cells, including the plasma membrane, endosomal and secretory vesicles, the cytoskeleton, mitochondria, lipid droplets, the cytoplasm and the nucleus (Gerke and Moss, 2002). Some of them are found extracellularly (AnxA1, AnxA2, and AnxA5) where they bind to different extracellular and cell membrane ligands and receptors that mediate systemic effects (Schloer et al., 2018). The biochemical properties of different annexins suggest they should be distributed in the cytoplasm of cells at resting Ca^2+^ levels, and moved to membranes when Ca^2+^ levels are elevated. Inside the cells, annexins are responsible for Ca^2+^-regulated endocytic and exocytic events, the maintenance and regulation of membrane-cytoskeleton contacts and membrane domain organization (Babiychuk and Draeger, 2000). For example, AnxA1 participates in the inward vesicle budding at late endosomes (Enrich et al., 2017). AnxA2 is involved in the regulation of actin dynamics at endosomes and in endosome maturation (Morel et al., 2009) and participates in late steps of Weibel-Palade bodies exocytosis in endothelial cells (Knop et al., 2004). In addition, AnxA8 is also related to post-endosomal trafficking events and is required for proper Weibel-Palade bodies maturation (Poeter et al., 2014). AnxA13 mediates the delivery of post-Golgi transport vesicles to the apical cell surface in polarized epithelial cells (Fiedler et al., 1995). Some annexins can also interact with the nucleus. In particular, AnxA11 plays a key role in the cytokinesis, the process by which the cytoplasm physically separates into two daughter cells during cell division (Tomas and Moss, 2003).

Annexin functions have also been linked to plasma membrane repair, an ongoing process observed in mechanically challenged tissues throughout the body. The functional role of AnxA1 in plasma membrane repair was demonstrated by interfering with AnxA1 availability or Ca^2+^ sensitivity, which inhibit mechanically induced membrane wound repair in HeLa cells (McNeil et al., 2006). Other annexins are also recruited to plasma membrane wound sites including AnxA2, A4, A5, A6, and A11 (Lennon et al., 2003; Bouter et al., 2011; Roostalu and Strähle, 2012; Boye et al., 2017). The recruitment of different annexins during membrane repair is of temporal order. For example, wound site recruitment of AnxA1 precedes that of AnxA2 in human endothelial cells (Koerdt and Gerke, 2017). In summary, annexins participate in many membrane processes, most of them occurring close to or at the plasma membrane.

**FIGURE 2:**
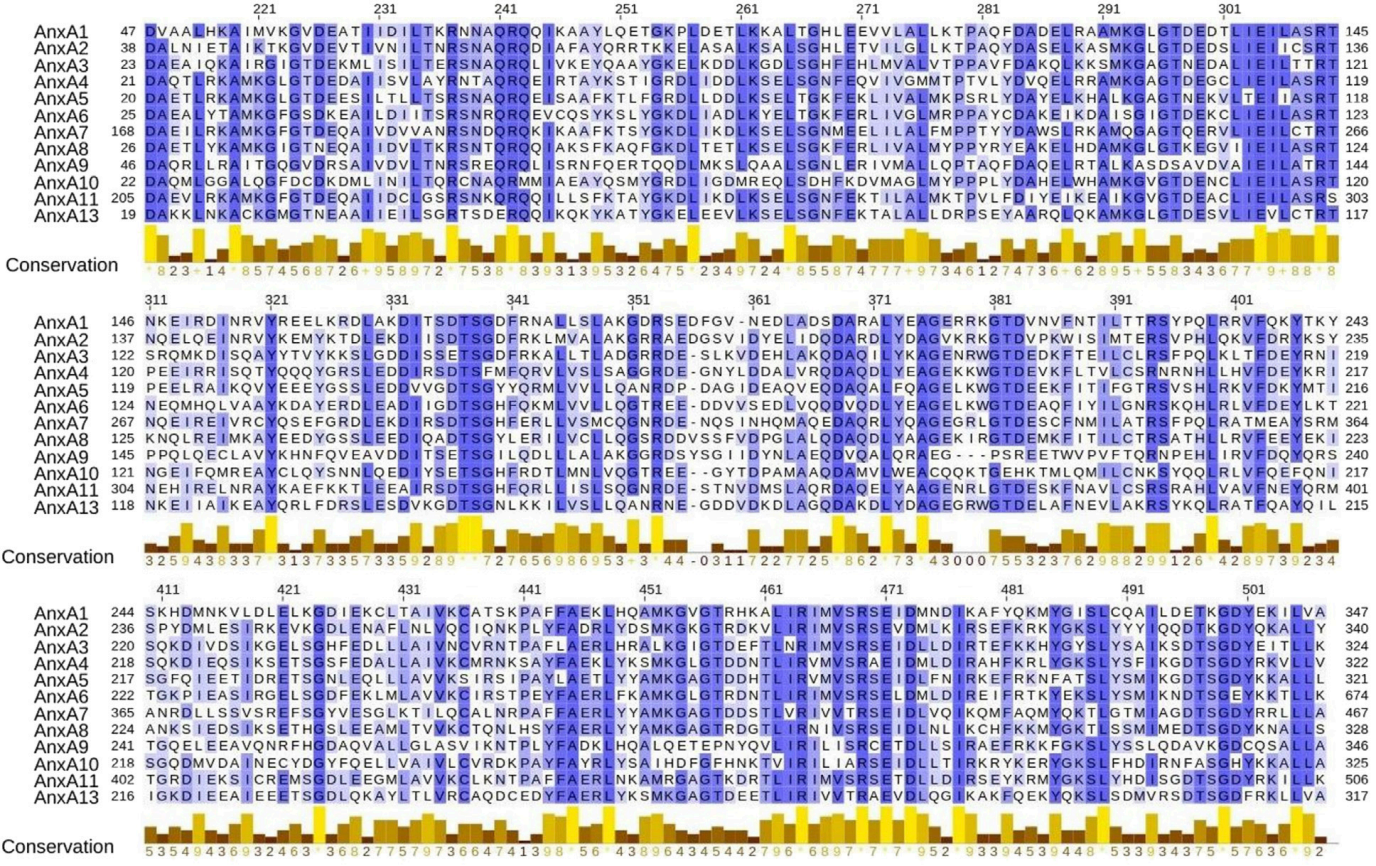
Protein sequence similarity of human AnxA1 to AnxA13. Multiple sequence alignment of annexins using amino acid complete sequences from canonical transcripts. For visualization purposes, the alignment is trimmed to show only the shared four domains. Amino acids are colored according to the percentage of identity. Conservation score of amino acid properties is shown per position. Amino acid sequences from proteins AnxA1 to AnxA13 where retrieved from Ensembl biomart, using GRCh38 human genome assembly and canonical transcripts. Multiple sequence alignment (MSA) was performed using Clustal Omega (Sievers et al., 2011). MSA was visualized, annotated with conservation score and trimmed to the region where all 12 shared the four domains using Jaview (Waterhouse et al., 2009).

Growing evidence suggests that annexins are involved in several diseases, including diabetes, cancer, autoimmune disorders and cardiovascular diseases (Grewal et al., 2021). In the last two decades, knockout (KO) mouse strains with targeted deletion of different annexins have been generated and have provided essential information on the function of each annexin (Grewal et al., 2016, 2021). All animals deficient in annexins are viable and developed entirely normal except for AnxA7, of which two different phenotypes have been described: first a lethal one due to cerebral hemorrhage (Srivastava et al., 1999) and later, a normal and viable one (Herr et al., 2001). The high degree of conservation (Figure 2) and the fact that most annexin-deficient mice are normal and viable suggests redundancy among annexin family members. However, wild type mice transplanted with annexin KO cells or annexin KO mice developed strong phenotypes under stress or pathological conditions (Grewal et al., 2016, 2021). In this context, the role of different annexins in the context of CVD has been highlighted in the last years.

### Annexin A1, a pro-resolving protein implicated in cardiovascular disease

Annexin A1 (AnxA1), the product of *ANXA1* gene and also known as lipocortin, is a 37 kDa protein that plays an important role in cardiovascular system, including its pro-resolving function in inflammation related to atherosclerosis and myocardial infarction (de Jong et al., 2017). This protein is expressed in several tissues such as heart, blood vessels, brain, adrenal glands and in many cells including endothelial and epithelial cells, although it is particularly abundant in cells of myeloid origin such as monocytes, macrophages and neutrophils (Gerke et al., 2005; D’Acunto et al., 2014). AnxA1 expression is regulated by glucocorticoids that increase *ANXA1* gene expression and also protein secretion from existing intracellular pools through protein kinase C activation (Gavins and Hickey, 2012). Indeed, glucocorticoid-induced leucine zipper has been shown to mediate AnxA1 upregulation at the promotor level through binding with the transcription factor PU.1 in neutrophils (Ricci et al., 2017). Within cells, AnxA1 is found in different locations, including the plasma membrane, endosomes, secretory vesicles, cytoskeleton and nucleus. AnxA1 can also be cleaved at the N-terminal domain by different proteases including metalloproteases and elastases among others (Rescher et al., 2006; Vong et al., 2007; Blume et al., 2012). AnxA1 exerts anti-inflammatory actions outside the cells (Perretti et al., 1996; Wein et al., 2004). Exogenous AnxA1 can also be cleaved by proteolytic enzymes leading to AnxA1-derived peptides such as Ac2-26 (Rescher et al., 2006). Both, AnxA1 and its derived peptide Ac2-26 bind to the formyl peptide receptors (FPRs), which are transmembrane G-protein-coupled receptors highly expressed in leukocytes (Walther et al., 2000). Although there are 3 different human FPRs, full length AnxA1 bind specifically to FPR2 (Perretti and D’Acquisto, 2009). However, AnxA1 derived peptides bind to both, FPR1 and FPR2 (Walther et al., 2000; Ernst et al., 2004). Both, AnxA1 and Ac2-26 exert their anti-inflammatory properties through FPR2 (Perretti et al., 2002; Dufton et al., 2010). For example, AnxA1 activates FPR2 to increase the release of anti-inflammatory cytokines such as IL-10 in human monocytes (Ferlazzo et al., 2003). In addition, Ac2-26 induces FPR2/FPR1 heterodimerization leading to activation of proapoptotic pathways in monocytes (Cooray et al., 2013).

AnxA1 is involved in controlling a variety of cellular activities such as membrane transport (endo/exocytosis), signal translation, actin dynamics, regulation of proliferation, differentiation, migration and apoptosis (Grewal et al., 2016). As commented, one of the most important functions of AnxA1 is its anti-inflammatory capacity. The first evidence was supported by analysis of AnxA1-deficient mice showing aberrant inflammation and complete resistance to the anti-inflammatory effects of glucocorticoids (Hannon et al., 2003). One of the main mechanisms is the regulation of leukocyte recruitment by AnxA1. Leukocytes move from the circulation to the injured tissue through the endothelium in a controlled sequence that include rolling, adhesion, endothelial transmigration, and chemotactic migration (Ley et al., 2007). To avoid chronic inflammatory response and excessive leukocyte recruitment, endogenous pro-resolving molecules such as lipid mediators (resolvins and protectins, among others) and peptides/proteins (AnxA1, galectins, melanocortins, among others) are released by different cell types to block leukocyte adhesion (Hajishengallis and Chavakis, 2013). For instance, AnxA1 and Ac2-26 are able to inhibit human neutrophils adhesion to activated endothelial cells under both static or flow conditions *in vitro* (Perretti et al., 1993; Hayhoe et al., 2006). The adhesion of neutrophils to the endothelium contributes to monocyte recruitment by depositing chemotactic proteins on the endothelium (Silvestre-Roig et al., 2020). The recruitment of monocytes is also inhibited by AnxA1 or Ac2-26 during acute inflammation (Getting et al., 1997).

Leukocyte adhesion to the injured endothelium is a critical step in atherosclerosis development. Since AnxA1 plays an important role in leukocyte recruitment, it might be a potential candidate to limit inflammation in atherosclerotic plaque development and progression and its derived CVD. In fact, AnxA1 polarized macrophages toward an anti-inflammatory phenotype (Li et al., 2011), which has been associated to beneficial/protective effects in atherosclerosis and CVD. AnxA1 are localized in macrophages and endothelial cells in human coronary atherosclerotic plaques (Bagnato et al., 2007; Drechsler et al., 2015). In addition, AnxA1 is also expressed in SMCs of human carotid atherosclerotic plaques and its expression is increased in asymptomatic patients compared to those have suffered a recent acute cerebrovascular event (Viiri et al., 2013). Several studies have demonstrated that AnxA1 and Ac2-26 protect from atherogenesis and atheroprogression in mice. Lack of either FPR2 or its ligand AnxA1 in ApoE-deficient mice accelerates atherosclerotic lesion formation. Aortic root lesions were larger in size and contained more leukocytes in AnxA1^−/−^ApoE^−/−^ double deficient mice compared to ApoE^−/−^ mice (Drechsler et al., 2015) (Table 1). In the other hand, intraperitoneal administration of recombinant AnxA1 to low-density receptor (LDLR) knockout mice fed a hyperlipidemic diet reduced neutrophil rolling and adhesion to endothelial cells, thereby delaying atherosclerotic plaque progression (Kusters et al., 2015). In addition, administration of Ac2-26 lowered lesional macrophages content as well as plaque size in ApoE-deficient mice (Drechsler et al., 2015). Ac2-26 counteracted chemokine-mediated integrin activation in monocytes and neutrophils, preventing leukocyte recruitment to atherosclerotic lesions (Drechsler et al., 2015). Similar results were observed when collagen IV-targeted nanoparticles containing Ac2-26 (Col IV-Ac2-26) were administrated to ApoE-deficient mice. Col IV-Ac2-26 decreased collagenase activity and ICAM-1 expression and increased *Col3a1* and *IL-10* mRNA expression, leading to a reduced necrotic core and lesion size in atherosclerotic plaques present in the aortic root (Fredman et al., 2015). AnxA1 is also protective in healing after wire injury in carotid arteries. Neointima expansion was aggravated in AnxA1^−/−^ApoE^−/−^ double deficient mice compared with ApoE^−/−^ mice, probably by accumulation and proliferation of macrophages (de Jong et al., 2017). The interaction between ATP binding cassette transport A1 (ABCA1) and AnxA1 has been proposed as an atheroprotective mechanism (Shen et al., 2020). ABCA1 is a membrane transporter that mediates cholesterol efflux in macrophages and exerts anti-inflammatory effect by binding to Apolipoprotein A-I (Tang et al., 2009). In resting conditions, neutrophils, monocytes and macrophages constitutively contain a large amount of AnxA1 in their cytoplasm (Morand et al., 1995). Upon leukocyte activation, AnxA1 is mobilized to the cell surface and secreted (Perretti et al., 1996). In particular, in macrophages, the interaction of ABCA1 with AnxA1 promotes the translocation of AnxA1 from the cytoplasm to the cell membrane, thus mediating AnxA1 secretion (Wein et al., 2004). Thereafter, AnxA1 can bind to FPR2 to induce its anti-inflammatory actions. Reciprocally, it has been suggested that AnxA1 may also increase ABCA1 expression and cholesterol efflux, favoring a regulatory loop between both proteins and reinforcing their anti-inflammatory actions (Chapman et al., 2003). However, this hypothesis needs to be investigated.

**TABLE 1:** Annexins and cardiovascular diseases.

Annexin	Cellular localization	Cellular function	Tissue distribution	Cardiovascular diseases	Disease model	Outcome	References
A1	Plasma membrane, cytoplasm, nucleus, secretory vesicles	Signal transduction, endo and exocytosis, cellular metabolism, proliferation, migration, differentiation, survival, and inflammation	Lung, kidney, bone marrow, intestine, spleen, thymus, brain, vascular and cardiac tissues, and seminal fluid	Atherosclerosis	ApoE/AnxA1 or ApoE/FPR2 double KO mice	⇑ Lesion size and leukocyte accumulation	Drechsler et al. (2015)
					ApoE KO mice treated with Ac2-26	⇓ Lesion size and leukocyte accumulation	Drechsler et al. (2015)
					LDLR KO mice treated with rAnxA1	⇓ Lesion size and necrotic core	Kusters et al. (2015)
					LDLR KO mice treated with Col IV-Ac2-26	⇓ Lesion size and collagenase activity	Fredman et al. (2015)
				Restenosis	ApoE/AnxA1 double KO Wire injury	Accelerated neointimal formation	de Jong et al. (2017a)
				Aortic Disection	Ac2-26 treatment in ApoE KO mice	⇓ Acute aortic disection	Zhou et al. (2022)
				Myocardial Infarction	AnxA1 KO mice	Increased infarct size, inflammation and fibrosis	(Qin et al., 2013, 2017)
					AnxA1 KO mice	⇓ cardiac funcionality	Ferraro et al. (2019)
					Rats treated with rAnxA1	⇓ Infarct size	D’Amico et al. (2000)
					Ac2-26 treatment in rats	⇓ Infarct size	La et al. (2001)
					Ac2-26 treatment in mice or rats	Rescued left ventricle function after ischemia	Qin et al. (2013)
					Overexpression in pigs	Improved cardiac funcionality	Ferraro et al. (2019)
				Stroke	Ac2-26 in MCAO model	⇓ Infarct size	Relton et al. (1991)
					IR injury in AnxA1 KO mice	⇑ Cerebral infarct volume	Gavins et al. (2007)
					rAnxA1 in MCAO model	⇓ Infarct size	Gavins et al. (2007)
					Ac2-26 after carotid ligation	⇓ Leukocyte adhesion to endothelium	Smith et al. (2015)
					I/R injury in WT mice treated with Ac2-26	⇓ Infarct volume and brain injury	Xu et al. (2021)
A2	Plasma membrane, cytosol, chromaffin granules	Membrane repair, trafficking, RNA export, cell growth, differentiation, apoptosis, and migration	Pancreas, lung, colon, ileum, and adrenal and vascular tissues	Atherosclerosis	ApoE/AnxA2 double KO mice	No effect	Hedhli et al. (2012)
					Carotid ligation in ApoE KO mice	⇓ atherosclerosis	Zhang et al. (2020)
				Stroke	rAnxA2 treatment in rat carotid artery thrombus model	Improved cerebral blood flow ⇓ Thrombus size	Ishii et al. (2001)
					Focal embolic stroke in rats treated with rAnxA2	⇓ Infarct size	Zhu et al. (2010)
					Middle cerebral artery embolization in rats treated with rAnxA2	⇓ Infarct size	Tanaka et al. (2007)
A5	Plasma membrane, Golgi, endoplasmic reticulum, late endosomes, phagosomes, mitochondria, nucleus, and cytoplasm	Cell cycle, differentiation, apoptosis and phagocytosis	All tissues, except neurons	Atherosclerosis	ApoE KO mice treated with rAnxA5	⇓ Plaque inflammation	Burgmaier et al. (2014)
						No effect in plaque size	
					ApoE KO mice treated with rAnxA5	⇓ Atherosclerotic burden and plaque size	Stöhr et al. (2017)
					ApoE*3-Leiden mice treated with rAnxA5	⇓ Neointima formation in femoral arteries	Ewing et al. (2012)
				Myocardial Infarction	ApoE*3-Leiden mice treated with rAnxA5	⇓ Infarct size and improved cardiac function	de Jong et al. (2018)
					C57BL6 mice treated with SDF1/AnxA5 fusion protein	⇓ Infarct size and improved cardiac function	Huang et al. (2019)
A7	Plasma membrane, secretory vesicles	GTPase activity, Ca^2+^ homeostasis, exocytic pathways, cell survival and growth	47 kDa isoform: All tissues, except skeletal muscle, 51 kDa isoform: brain, heart, and myotubes	Atherosclerosis	ApoE KO mice treated with 6-amino-2, 3-dihydro-3-hydroxymethyl-1, 4-benzoxazine	⇓ Lesion size and macrophages content	Li et al. (2013)

AnxA1 plays an important role in maintaining SMCs homeostasis and protection against acute aortic dissection. Thus, AnxA1 deficiency in SMCs aggravates acute aortic dissection (AAD) in mice (Zhou et al., 2022). In addition, Ac2-26 administration decreased AAD incidence in AnxA1^−/−^ mice decreasing SMCs phenotypic switching to a secretory phenotype (Zhou et al., 2022). Taken together, *in vivo* studies reveal that administration of AnxA1 or derived peptides could serve to prevent pathological vascular remodeling.

AnxA1 and its derived peptides has both been shown to be cardioprotective in mouse and rat models of myocardial infarction. AnxA1-deficient mice showed a reduced cardiac functionality, increased macrophages content in the ischemic area (Ferraro et al., 2019) and increased infarct size (Qin et al., 2013, 2017). Recombinant AnxA1 administration reduced the extent of infarct size in a model of myocardial ischemia reperfusion in rats (D’Amico et al., 2000). The effect of AnxA1 was related to a reduction in tumor necrosis factor-α (TNF-α) and macrophage inflammatory protein-1α expression, as well as myeloperoxidase (MPO) activity, leading to reduced leukocyte extravasation into cardiac tissues (D’Amico et al., 2000). In addition, Ac2-26 administration decreased infarct size and diminished MPO activity and IL-1β levels in an experimental model of rat myocardial ischemia/reperfusion injury (La et al., 2001). Ac2-26 treatment preserved cardiomyocyte contractile function and decreased cardiac myocyte injury through activation of p38-MAPK, PKC, and ATP-sensitive potassium channels (Ritchie et al., 2005). Exogenous administration of Ac2-26 also increased cardiomyocyte viability and rescued left ventricle function after ischemic injury (Qin et al., 2013). Full-length AnxA1 overexpression also showed cardioprotective effects in superior animals such as pigs (Ferraro et al., 2019). Unfortunately, all these studies are based in models of acute damage after reperfusion and there are no studies linking AnxA1 and myocardial repair.

Finally, the role of AnxA1 has been also analyzed in models of ischemic stroke. Intracerebroventricular administration of Ac2-26 reduced infarct size and cerebral edema in the middle cerebral artery occlusion model (MCAO) in rats (Relton et al., 1991). Targeting FRP2 receptors with Ac2-26 also decreased leukocyte adhesion to endothelium in a model of bilateral common carotid artery occlusion in mice (Smith et al., 2015). Similar protective effect was observed using recombinant AnxA1 (Gavins et al., 2007). In addition, AnxA1-deficient mice showed more white blood cell adhesion in cerebral venules than wild type mice, and exhibited a higher cerebral infarct volume and worse neurological score in the MCAO model (Gavins et al., 2007). This effect was further confirmed and related to activation of 5′ adenosine monophosphate-activated protein kinase and inhibition of mammalian target of rapamycin (mTOR) (Xu et al., 2021).

On the whole, AnxA1 has vasculo- and cardio-protective effects through mechanisms involving mainly inhibition of leukocyte extravasation. The therapeutic potential of AnxA1 and Ac2-26 has been well established in experimental models of CVD, but their translation to human is awaiting.

### Annexin A2, an anti-thrombotic protein

Annexin A2 (AnxA2), also known as lipocortin II or calpatin-1 heavy chain, is the product of *ANXA2* gene that is traduced to a 36 kDa protein. As other members of the family, AnxA2 is a Ca^2+^-dependent phospholipid-binding protein, but it can also bind and bundle to actin filaments (Ikebuchi and Waisman, 1990). AnxA2 is expressed by endothelial cells, monocytes/macrophages, some tumor cells (Gerke et al., 2005), and in a wide variety of tissues, being its expression different depending on the tissue analyzed. It is highly expressed in the pancreas, lung, ileum, colon, and adrenal tissues, while low AnxA2 expression are found in liver, kidney, testis, and spleen (Seidah et al., 2012). AnxA2 regulates microdomain formation and membrane repair, trafficking along endo- and exocytic pathways, and RNA export from the nucleus (Bharadwaj et al., 2013). At the cellular level, AnxA2 participates in cell growth, migration, differentiation, and apoptosis (Rescher and Gerke, 2004; Bharadwaj et al., 2013).

The N-terminal domain of AnxA2 can bind and form a heterotetramer with p11 (S100A10), which can be translocated from the cytoplasm to the outer membrane leaflet (Réty et al., 1999). Extracellular p11/AnxA2 can enhance the activation of plasmin and vascular fibrinolysis by binding to plasminogen and tissue plasminogen activator in endothelial cells (Hajjar et al., 1994; Hajjar, 2015). Different animal studies support the role of AnxA2 in fibrin clearance. AnxA2-deficient mice display a normal phenotype but show substantial accumulation of fibrin in several tissues such as kidney, lungs, and spleen, among others (Ling et al., 2004). In addition, thrombotic occlusion is increased after experimental injury to the carotid artery in AnxA2-deficient mice (Ling et al., 2004). Similarly, S100A10-deficient mice show reduced clearance of thrombi (Surette et al., 2011). Accordingly, AnxA2-deficient mice have reduced S100A10 levels probably due to increase in S100A10 ubiquitination and degradation (He et al., 2008).

AnxA2 also participates in angiogenesis, a key contributor to atherosclerosis progression (Sedding et al., 2018). It has been demonstrated that AnxA2-deficient mice exhibit defects in new blood vessel formation (Ling et al., 2004). In addition, overexpression of AnxA2 receptor inhibited cell proliferation and migration as well as tube formation in endothelial cells, and neovascularization in mouse matrigel assays (Guo et al., 2018). Despite the relationship of AnxA2 with angiogenesis and thrombosis, its relation with atherosclerosis is controversial. Endothelial cell apoptosis plays a key role in the development of atherosclerosis. In this sense, silencing of AnxA2 upregulates caspase activity and increases apoptosis in human endothelial cells (Jiang et al., 2015). However, it is unknown the effect of AnxA2-deficiency in other cell types implicated in atherosclerosis. It has been shown that plasmin/plasminogen signaling in human monocytes utilizes p11/AnxA2 as a receptor and triggers signaling through JAK/STAT, Akt-dependent NF-kB activation, as well as ERK1/2 and p38MAPK, leading to the induction of proinflammatory genes and inflammatory cell recruitment (Li et al., 2007). Furthermore, AnxA2 has been implicated in SMCs migration (Won et al., 2011). *In vitro* migration assays have shown that AnxA2 overexpression or inhibition increases or decreases, respectively, the migration of rat SMCs migration after platelet-derived growth factor stimulation (Won et al., 2011). Accordingly, AnxA2 expression was increased in vascular neointima of carotid artery after balloon injury (Won et al., 2011). AnxA2 expression is also markedly upregulated in atherosclerotic lesions of ApoE KO mice (Wang et al., 2018). However, germline deletion of AnxA2 did not reduce atherosclerosis burden in ApoE-deficient mice (Hedhli et al., 2012) (Table 1). Conversely, AnxA2 deficiency suppressed atherogenic integrin α5 signaling induced by oscillary shear stress in a partial carotid ligation model of atherosclerosis in ApoE deficient mice (Zhang et al., 2020).

Elevated levels of low-density lipoprotein cholesterol (LDL-c) are currently considered to be the primary risk factor for the development of atherosclerosis. LDLR expression contributes to the plasma LDL-c levels and increased LDLR expression may reduce LDL-c concentrations. The serin protease proprotein convertase subtilisin/kexin-9 (PCSK9) is mainly produced in the liver and is essential for metabolism of LDL-c by inhibiting LDLR recirculation to the cell surface with the consequent upregulation of LDLR-dependent LDL-c levels (Abifadel et al., 2003). Importantly, AnxA2 is able to inhibit PCSK9-mediated LDLR downregulation, leading to elevated LDLR levels (Mayer et al., 2008; Seidah et al., 2012). Untreated AnxA2-deficient mice exhibited high levels of circulating PCSK9 and LDL-c without affecting HDL-c, and adenoviral overexpression of AnxA2 in mouse liver increased LDLR protein levels in mice (Seidah et al., 2012). In recent years, *ANXA2* gene variants have also been identified through single nucleotide polymorphism analysis that directly influence circulating LDL-c levels, supporting AnxA2 levels as a potential therapeutic target for reducing LDL-c concentrations (Fairoozy et al., 2017).

AnxA2 also plays an important role in ischemic stroke. As commented previously, AnxA2/p11 complex serves as an assembly site for plasminogen and tissue plasminogen activator (tPA) (Dassah et al., 2009; Madureira et al., 2011). Treatment with recombinant AnxA2 decreased the thrombolytic effect of tPA in a rat model of focal ischemic stroke (Zhu et al., 2010). Animals receiving both, recombinant AnxA2 and tPA had reduced brain hemorrhage and infarct volume and greater cerebral blood flow compared to those treated with tPA alone (Zhu et al., 2010). Similarly, treatment with recombinant AnxA2 improved blood flow and reduced infarct size in a model of middle cerebral artery embolization in rats (Tanaka et al., 2007). Administration of recombinant AnxA2 was also associated with improved cerebral blood flow and reduced thrombus size in a rat carotid artery thrombus (Ishii et al., 2001). In summary, AnxA2 could impact 3 main mechanisms involved in CVD, such as angiogenesis, thrombosis and lipid metabolism. Moreover, enhancement of fibrinolytic and hypolipemic activity by AnxA2 could modulate the hypercoagulable and hyperlipidemic state of CVD.

### Annexin A3, a protein implicated in myocardial infarction

Annexin A3 (AnxA3), also known as lipocortin III and placental anticoagulant protein II, is expressed in many cell types and different tissues such as heart, spleen, brain, lung, liver and, to a greater extent, in adipose tissue (Watanabe et al., 2012; Meadows and Cleaver, 2015; Pan et al., 2015; Du et al., 2018). AnxA3 is implicated in different cell functions such as cell migration and differentiation, inflammatory responses, cell signaling, membrane transport, and cytoskeletal interactions (Hofmann et al., 2000; Park et al., 2005; Meadows and Cleaver, 2015; Huang et al., 2020). This protein has been related with devasting pathologies such as cancer (Yang et al., 2021). However, although AnxA3-deficient mice are viable, the lack of animal models using these deficient animals has not allowed us to know the role of AnxA3 in CVD. *In vitro* experiments have demonstrated that conditioned media from HEK 293 cells overexpressing AnxA3 induces migration of endothelial cells (Park et al., 2005). In addition, VEGF increases AnxA3 expression in endothelial cells, and AnxA3 siRNA-treated human umbilical vein endothelial cells have a reduced migration ability in wound-healing assays (Park et al., 2005). Conditional inhibition of AnxA3 in mouse vascular endothelium (AnxA3f/f; Tie2-Cre) has shown that loss of this protein is not essential for embryonic blood vessel formation but is necessary for the parallel alignment of arteries and veins, which is essential for proper blood flow and vessel function (Huang et al., 2020). AnxA3 is also highly expressed in myocardial cells during acute myocardial infarction and *in vivo* AnxA3 inhibition using a short hairpain RNA in rats reduced infarct size, inflammatory response, *α*-actin, collagen type I and III by activation of the PI3K/Akt signaling pathway (Meng et al., 2019).

### Annexin A5, more than an apoptosis marker

Annexin A5 (AnxA5) is a 36 kDa protein and is the most abundant annexin in all cells and tissues, except neurons. It is predominantly expressed as an intracellular protein although it is also found in cerebrospinal fluid, plasma, and urine (Boersma et al., 2005). In the cells, AnxA5 is localized in nucleus, Golgi, endoplasmic reticulum, late endosomes, mitochondria, phagosomes and plasma membrane (Gerke et al., 2005; Ghislat et al., 2012). At the functional level, AnxA5 has been related to membrane trafficking, Ca^2+^ signaling, phagocytosis, cell cycle, growth and apoptosis (Hawkins et al., 2002; Wang et al., 2003; Monastyrskaya et al., 2007; Faria et al., 2011; Ghislat et al., 2012). Due to its preferential phosphatidylserine (PS) binding property, AnxA5 has been used as a marker for detection of cells undergoing apoptosis (Koopman et al., 1994). The exposure of PS on the cell surface of dying cells induced tissue factor activation and thrombin generation. In this regard, AnxA5 appears to play an important role in blood coagulation as it competes with prothrombin for binding PS and inhibits phospholipase A1 activity (Tzima et al., 2000). Given its ability to detect apoptotic cells, AnxA5 is a powerful tool for identifying atherosclerotic plaques. In this regard, radiolabeled AnxA5 has been used for noninvasive detection of apoptosis in both experimental and human carotid atherosclerosis (Kolodgie et al., 2003; Kietselaer et al., 2004). In addition, when ^18^F-FDG, a marker of inflammation, was compared with 99mTc-labeled to detect atherosclerotic plaques in ApoE-deficient mice, it was observed that ^18^F-FDG was better than 99mTc-AnxA5 at detecting atherosclerotic lesions, but 99mTc-AnxA5 was better than ^18^F-FDG at detecting advanced lesions, indicating that AnxA5 could be as useful indicator of plaque vulnerability (Zhao et al., 2007).

Therapeutically, administration of recombinant AnxA5 reduced plaque inflammation but not plaque size of advanced lesions in ApoE-deficient mice by interfering with monocyte recruitment and activation at the inflamed site (Burgmaier et al., 2014) (Table 1). *In vitro* experiments demonstrated that AnxA5 decreased TNF-α and increased IL-10 in macrophages, suggesting a polarization of macrophages toward an anti-inflammatory phenotype (Burgmaier et al., 2014). In contrast, AnxA5 reduced atherosclerotic burden and plaque size in ApoE-deficient mice when administered prior to plaque development (Stöhr et al., 2017). Interestingly, AnxA5 inhibited the uptake of oxidized-LDL by macrophages, delaying foam cell formation and thus exerting an atheroprotective effect (Domeij et al., 2013). Systemic administration of AnxA5 also reduced neointima formation in femoral arteries after perivascular cuff-mediated injury model in ApoE*3-Leiden mice (Ewing et al., 2012). In addition, The GENDER study investigated the association between AnxA5 single nucleotide polymorphisms (SNPs) and the risk of restenosis in patients undergoing percutaneous coronary intervention (PCI). The allelic association test identified two SNPs, rs4833229 and rs6830321, which were significantly associated with restenosis after percutaneous angioplasty. These data suggest that AnxA5 genotype functions as risk marker for restenosis (Ewing et al., 2012).

The effect of AnxA5 treatment on left ventricular function and remodeling after myocardial ischemia-reperfusion injury was studied in hypercholesterolemic ApoE*3-Leiden mice (de Jong et al., 2018). By suppression of the inflammatory response and cardiac macrophage content, AnxA5 administration attenuated long term adverse effects of left ventricle remodeling, reduced infarct size and improved cardiac function (de Jong et al., 2018). Finally, treatment with a fusion protein between AnxA5 and stromal-derived factor 1, a known cytokine that protects heart from ischemic injury, reduced infarct size and improved cardiac function after myocardial infarction in mice (Huang et al., 2019).

In summary, AnxA5 is an imaging tool for advanced atherosclerotic plaques in relation to its capacity to detect apoptotic cells. Moreover, both vascular and cardiac-protective effects have been demonstrated for AnxA5 in experimental models of CVD.

### Regulation of cardiac function by Annexin 6

Annexin A6 (AnxA6) is a 68 kDa protein that is ubiquitously and highly expressed, except in the colon, epithelial cells of the small intestine and parathyroid gland (Enrich et al., 2011). Unlike the other annexins, AnxA6 contains two annexin cores composed of four annexins repeats each connected by a linker located between the fourth and fifth annexin repeats. In addition, alternative splicing generates two different isoforms of AnxA6, named A6-1 and A6-2. A6-1 is present in most cells and tissues but A6-2 is upregulated in some transformed cells (Kaetzel et al., 1994). Like other Anxs, AnxA6 binds to phospholipids in cell membranes in a reversible fashion. In addition, AnxA6 is also localized in mitochondria and hepatic lipid droplets (Turró et al., 2006; Chlystun et al., 2013). The targeting of AnxA6 to membranes is regulated by the pH and the levels of cholesterol (de Diego et al., 2002). AnxA6 is implicated in the regulation of microdomain organization, cholesterol homeostasis, endocytic transport, cytoskeleton rearrangements, and stress response (Enrich et al., 2011). In addition, AnxA6 participates in cell growth, motility and differentiation as well as lipid and glucose homeostasis (Enrich et al., 2011, 2011; Chlystun et al., 2013; Grewal et al., 2016, 2017). AnxA6 is also connected with cardiac function. AnxA6 is strongly expressed in the heart and mice overexpressing AnxA6 have enlarged dilated hearts with lymphocytic infiltration and severe fibrosis, acute diffuse myocarditis, and mild fibrosis in pulmonary veins (Gunteski-Hamblin et al., 1996). Isolated cardiomyocytes overexpressing AnxA6 exhibit reduced basal Ca2+ levels and Ca2+ spike amplitude, leading to concomitant changes in contractility. In contrast, cardiomyocytes from AnxA6-deficient mice showed an increase in contractility and accelerated diastolic Ca^2+^ removal from the cytoplasm (Song et al., 2002). However, Anx6-deficiency was not associated with alterations in blood pressure and heart rate compared to wild type mice (Hawkins et al., 1999).

Although the role of AnxA6 in the development of vascular damage is currently unknown, this protein has been linked to vascular calcification, a process that increases plaque instability and accelerates aortic valve stenosis (Ruiz et al., 2015). Matrix vesicles (MV), secreted by SMCs, form the first nidus of mineralization (Martin-Ventura et al., 2022). MV cargo can be modified under procalcifying or pro-inflammatory environment, resulting in a reduction of calcification inhibitors in MV and their enrichment in phospatidylserine, CD63 and AnxA6 (Kapustin et al., 2015). In addition, small interfering RNA inhibition of AnxA6 expression reduced SMCs mineralization (Kapustin et al., 2011). AnxA6 is abundant at sites of vascular calcification in human atherosclerotic plaques (Kapustin et al., 2011) and also in calcified aortic valve (Cui et al., 2017).

### Annexin A7, an annexin with GTPase activity

AnxA7, also named synexin, presents two isoforms: one of 47 kDa, which is basically expressed in all tissues except skeletal muscle, and another isoform of 51 kDa, which is found in cardiac tissue, brain and myotubules (Selbert et al., 1995). This protein is principally associated with the nuclear envelope, secretory vesicle, and plasma membrane in a Ca^2+^-dependent manner (Kuijpers et al., 1992). AnxA7 has GTPase activity, facilitates PKC activation, and contributes to Ca^2+^ homeostasis and the regulation of exocytic pathways (Caohuy and Pollard, 2002; Taniuchi et al., 2012), as well as prostaglandin production (Luo et al., 2015). In addition, it has demonstrated that AnxA7 is an endogenous inhibitor of phosphatidylcholine-specific phospholipase C (PC-PLC) (Li et al., 2013). AnxA7 has been proposed to be involved in DNA damage repair, tumorigenesis and cell senescence (Srivastava et al., 2003; Leighton et al., 2017).

As mentioned before, two different phenotypes have been described for AnxA7-deficient mice. The first knockout of AnxA7 showed a lethal phenotype induced by cerebral hemorrhage at embryonic day 10 (Srivastava et al., 1999). Heterozygous AnxA7 mice were viable but with a substantial defect in insulin secretion and alterations in Ca^2+^ signaling by a reduction in inositol 1,4,5-trisphosphate receptor expression and function in pancreatic islets (Srivastava et al., 1999). In contrast, the second generated AnxA7-deficient mice was viable with no apparent defects (Herr et al., 2001). The discrepancy could be due to differences in the induced mutations, design and integration sites of the targeting construct, with possible altered expression of genes near the integration site, and different genetic background.

In terms of vascular remodeling, AnxA7 has been associated with atherosclerosis development. Treatment of atheroprone ApoE-deficient mice with 6-amino-2, 3-dihydro- 3-hydroxymethyl-1, 4-benzoxazine (ABO), an inhibitor of AnxA7 GTPase, reduced atherosclerotic burden and plaque size compared with untreated mice (Li et al., 2013). This reduction was accompanied by a decrease in lipid deposition, necrotic core size, and pro-inflammatory macrophages content, as well as an increase in anti-inflammatory macrophages, collagen content and SMCs in atherosclerotic lesions, which increased plaque stability. Reduced AnxA7 GTPase activity is also involved in ABO-induced autophagy and inhibited apoptosis in cultured endothelial cells and in aortic endothelium of ApoE-deficient mice (Wang et al., 2010; Li et al., 2013), probably through increased homeobox containing 1 (HMBOX1) expression (Ma et al., 2016). In this context, low levels of HMBOX1 has been related with endothelial cell apoptosis and atherosclerosis progression (Ma et al., 2015, 2016).

AnxA7 is also highly expressed in the heart. Based on the studies in isolated cardiomyocytes from viable AnxA7-deficient mice, it has been shown that AnxA7 deficiency induces misfunctioning of the apparatus responsible for cardiac contraction, probably due to defects in Ca^2+^ homeostasis (Herr et al., 2001). In addition, AnxA7 can interact with ryanodine receptor and sorcin, which are involved in the coupling of calcium channels to the contractile machinery of cardiac muscle (Verzili et al., 2000). AnxA7 deficiency induced an increase in heart/body weight ratio in a transverse aortic constriction pressure overload model, which induces cardiac hypertrophy (Voelkl et al., 2014). This elevated ratio observed in AnxA7-deficient mice was associated with increased expression of several genes, probably induced by the Ca^2+^-regulated cardiac nuclear factor of activated T cells (NFAT) in an AnxA7-dependent manner (Voelkl et al., 2014).

### Annexin A8 and endothelial function

AnxA8, also known as vascular anticoagulant-beta 1 (VAC-β), is expressed at low levels in several tissues such as kidney, skin, placenta, liver, cornea, and lungs (Sarkar et al., 1994; Stein et al., 2005; Runkel et al., 2006). Until now, the role of AnxA8 is poorly characterized but it has been implicated in different cell functions. AnxA8 regulates proliferation of endometrial cells through the Akt signaling pathway (Jiang et al., 2019), effect that could be related with miR185-3p expression. In this respect, inhibition of miR185-3p promotes AnxA8 expression while increased miR185-3p levels reduce AnxA8 expression and inhibit proliferation in cervical cancer cells (Zhang and Han, 2021). In addition, overexpression of miR140-3p inhibit cell proliferation, migration, invasion and endothelial-mesenchymal transition *via* targeting AnxA8, effect reverted by taurine-upregulated gene 1 (Yuan et al., 2021). AnxA8 also regulates the phenotypic plasticity of retinal epithelial cells (Lueck et al., 2020). AnxA8 promotes VEGF-A driven endothelial cell sprouting (Heitzig et al., 2017) and is required to stabilize P-selectin on the endothelial surface through CD63 (Poeter et al., 2014). The delivery of CD63 to the cell surface is strongly reduced in AnxA8-deficient endothelial cells and correlates with a diminution of leukocyte adhesiveness under pro-inflammatory conditions in endothelial venules of AnxA8-deficient mice (Poeter et al., 2014). Given that leukocyte adhesion and extravasation to the arterial wall is a key process in the development of atherosclerotic plaque, it is possible that AnxA8 may play an important role in the development and progression of atherosclerotic lesions, although this hypothesis needs to be tested.

## Conclusion

In the last years, substantial progress has been made in research of annexins. Although their main function is to control membrane trafficking and repair, annexins can play a relevant role in the vascular remodeling that underlies the development of CVD. Different experimental models of loss- or gain-of-function in mice or treatment with recombinant annexins or specific annexin inhibitors have revealed the potential role of these proteins in CVD. Some of them should serve as diagnostic or therapeutic targets for CVD. For example, the affinity of AnxA5 for phosphatidylserine in apoptotic cells should make it possible to use this feature for diagnostic or therapeutic purposes. In addition, AnxA1, A2, A6, and A7 should serve as therapeutic targets for CVD. Different animal models have demonstrated the beneficial effects of these annexins for the treatment of atherosclerosis, restenosis, myocardial infarction or stroke. Peptide- or protein-based approaches may be suitable for *in vivo* administration. AnxA1 acting as an FPR2 agonist has potential therapeutic implications in the context of the inflammatory response underlying vascular remodeling. AnxA2 is relevant to control fibrinolysis and angiogenesis. Drugs that inhibit AnxA7 GTPase activity may hold promise for the treatment of CVD. Studies with recombinant proteins or specific inhibitors provide important proof of concept to promote further investigation of the exciting biology of annexins and highlight the potential for developing therapies that modulate annexins expression to limit CVD progression. However, the role of annexins in cardiovascular disease is not completely understood, and many questions need to be answered. Mainly AnxA1, A2, A5, and A7 have been shown to be involved in CVD, but could other annexins be related to CVD? The beneficial effect of AnxA1 is through the FPR2 receptor, but could FPR2 agonists represent a strategy to augment inflammatory resolution in CVD? Given that annexins have similar protein domain organization and, in some cases, overlapping functions, could inhibition/overexpression of more than one annexin provide a beneficial effect in preventing CVD? Benzoxazine derivate reduces lesion size in atheroprone mice, but could AnxA7 inhibitors be beneficial in myocardial infarction or stroke? In the near future, these and other potential questions could help to understand the role of annexins in the different cardiovascular pathologies. A deep understanding of the mechanisms of action of annexins and the controlled modulation of annexin expression/activity using different strategies such as small pharmacological molecules, delivery of stable recombinant proteins, targeted nanoparticles, adeno-associated virus overexpressing annexins, among others, could offer opportunities for therapy of pathological cardiovascular remodeling.
